# Assessing sleep among the next generation of healthcare delivery professionals

**DOI:** 10.5935/1984-0063.20190129

**Published:** 2020

**Authors:** Raissa Rossener, Isabella Rocha Morais, Maria Fernanda Caldeira, Sergio Tufik, Helena Hachul

**Affiliations:** 1 FICSAE - Faculdade Israelita de Ciencias da Saúde Albert Einstein, Saude da Mulher - São Paulo - SP - Brazil.; 2 UNIFESP, Psicobiologia - São Paulo - SP - Brazil.

Recently, Barahona-Correa et al.^1^ published an article entitled: “*Sleep disturbances, academic performance, depressive symptoms and substance use among medical students in Bogota, Colombia*”. It was a cross-sectional study with medical students from Pontificia Universidad Javeriana. The aim of the study was to evaluate sleep disturbances among medical undergraduates and the possible associations with depressive symptoms, academic achievement and substance use. The authors applied the *Pittsburgh Sleep Quality Index* (PSQI), the *Epworth Sleepiness Scale* (ESS), the *Berlin Questionnaire* (BQ) and *the Diagnostic and Statistical Manual of Mental Disorders - IV* and assessed use of both stimulants and tranquilizers. The results demonstrated that poor sleep quality is very common amongst the students and is associated with anxiety, depressive mood, reduced academic performance, daytime sleepiness, obstructive sleep apnea and use of psychotropic substances.

This is an interesting article on an important theme, as the quality of life of medical students and health professionals has a close association with the quality of care delivered. Therefore, we are writing to suggest other areas that could be evaluated in future to further enrich the outcomes and possible benefits of the current study in general, and particularly in relation to factors that affect women.

Sleep is one of the physical components of quality of life and is important to mental, physical and social well-being^2^. According to Marques et al.^3^, among college students poor sleep quality is intrinsically related to a poor quality of life. As Barahona-Correa et al.^1^ found, sleep disturbances are more common in medical students than in the general population, and they have a high rate of poor sleep quality (65.7%). According to a number of other studies, this can lead to reduced concentration and motivation, impaired decision-making and leadership capacities4, important attributes during graduation.

The article reported a high frequency of students who fulfilled the criteria for major depression (26%). A Brazilian study also demonstrated an increased number of mental health and sleep problems among medical students, including stress, anxiety, depression, low sleep quality and excessive daytime sleepiness^5^. These conditions can be correlated to the presence of insomnia, which could be evaluated using specific questionnaires such as the Insomnia Severity Index. Insomnia is a prevalent sleep disturbance^6^, especially in women, who generally report more sleep problems than men^7^^,^^8^.

It is, therefore, important to analyze the influence of gynecological factors on medical students’ sleep quality. Poor sleep quality is usually related to different factors in women’s lives, such as pregnancy, menopause and menstruation^8^, which are related to changes in the levels of ovarian hormones. Premenstrual syndrome (PMS) and the use of hormonal contraception are also variables that impact their sleep quality and functionality^9^^,^^10^. Women who suffered from PMS reported poor perceived sleep quality in an epidemiological study from São Paulo, Brazil, while women who used hormonal contraceptives had better sleep quality when compared to those who did not use these methods^10^. Although no differences between genders were observed in the study^1^, these gynecological factors can influence medical students’ sleep quality and should be taken into account to help healthcare professionals and medical courses to understand the differences between men and women during graduation and how gynecological factors can impact so many aspects of women’s lives.

We congratulate the authors on their study, since it can lead to important measures aimed at mitigating the issues encountered. Collectively, exploring these areas in future, studies will allow sleep and the possible negative effects of poor sleep quality, to be explored in even details in the next generation of healthcare professionals.
